# Recent Advances in Electrical Doping of 2D Semiconductor Materials: Methods, Analyses, and Applications

**DOI:** 10.3390/nano11040832

**Published:** 2021-03-24

**Authors:** Hocheon Yoo, Keun Heo, Md. Hasan Raza Ansari, Seongjae Cho

**Affiliations:** 1 Department of Electronic Engineering, Gachon University, 1342 Seongnamdaero, Sujeong-gu, Seongnam-si, Gyeonggi-do 13120, Korea; hyoo@gachon.ac.kr (H.Y.); hasanrazaadnan@gmail.com (M.H.R.A.); 2 Graduate School of IT Convergence Engineering, Gachon University, 1342 Seongnamdaero, Sujeong-gu, Seongnam-si, Gyeonggi-do 13120, Korea; 3 Department of Semiconductor Science & Technology, Jeonbuk National University, Jeonju-si, Jeollabuk-do 54896, Korea; kheo@jbnu.ac.kr

**Keywords:** electrical doping, 2D semiconductor material, transition metal dichalcogenide, graphene, atomically thin film

## Abstract

Two-dimensional materials have garnered interest from the perspectives of physics, materials, and applied electronics owing to their outstanding physical and chemical properties. Advances in exfoliation and synthesis technologies have enabled preparation and electrical characterization of various atomically thin films of semiconductor transition metal dichalcogenides (TMDs). Their two-dimensional structures and electromagnetic spectra coupled to bandgaps in the visible region indicate their suitability for digital electronics and optoelectronics. To further expand the potential applications of these two-dimensional semiconductor materials, technologies capable of precisely controlling the electrical properties of the material are essential. Doping has been traditionally used to effectively change the electrical and electronic properties of materials through relatively simple processes. To change the electrical properties, substances that can donate or remove electrons are added. Doping of atomically thin two-dimensional semiconductor materials is similar to that used for silicon but has a slightly different mechanism. Three main methods with different characteristics and slightly different principles are generally used. This review presents an overview of various advanced doping techniques based on the substitutional, chemical, and charge transfer molecular doping strategies of graphene and TMDs, which are the representative 2D semiconductor materials.

## 1. Introduction

Doping is the most common and feasible method used to control the properties of conventional semiconductors. However, 2D materials pose challenges owing to nonconformity to traditional doping techniques, such as ion implantation, which could damage the crystal structures of these materials [1,2,3]. Hence, sophisticated doping techniques are required to preserve the structure and intrinsic properties of 2D materials as well as modulate them according to required applications. Compared to other techniques, such as substitutional, electrical/magnetic field, and strain-effect-induced doping, organic molecular doping methods have numerous advantages. Organic compounds are widely available in natural and synthetic forms; they mostly consist of polymers that are repeated units of monomers. These organic compounds are viable candidates for inducing the required modulations in 2D materials owing to their easy availability and simplicity of doping. The soaking and dipping methods are commonly applied to 2D materials to induce doping effects using organic molecules. Self-assembled monolayers (SAM) play a major part in organic molecule-based doping techniques, which is why most recent organic-molecule-based doping studies have focused on utilizing SAM for doping [4,5]. SAMs were first discovered in 1978 and have since then been extensively used for salinization and surface functionalization of substrates. SAMs are based on silane or thiol coupling agents [6]. However, most recent studies have focused on silane-based SAMs. The dipole moments of end-functional groups in SAMs are normally responsible for inducing n- or p-type doping effects in 2D materials. However, one caveat to the application of SAMs is their hydrophilic nature, which renders the doping effects unstable after few days. Conversely, many organic molecules, such as monoethanolamine (MEA), benzyl viologen (BV), and SAM-thiol, have shown good stability of doping after a few days owing to their hydrophilic nature [6,7]. Nonetheless, organic-molecular-based doping techniques enable enhancement and modulation of the electrical, optical, mechanical, and flexible properties of 2D material-based devices. Using organic molecular doping techniques, many studies have shown unique applications for 2D materials, including photodetectors, photodiodes, p-n junctions, and gas/bio sensors [7,8]. Low-dimensional electronics can be realized even by group-IV atomic membranes, silicene and germane which are considered to be sp^2^-hybridized layers with honeycomb lattice structures. Thus, the doping techniques can be further extended into those new functional materials with wide applications owing to their higher Si processing compatibility and flexibility. In this work, we have compiled some recent studies that have utilized organic molecules as dopants for 2D materials, with a particular interest in graphene and transition metal dichalcogenides (TMDs) by which numerous device applications have been reported. 

## 2. Graphene Doping

### 2.1. Background

In 2004, Drs. Geim and Novoselov of the University of Manchester, UK, succeeded in exfoliating graphene from bulk graphite using the scotch tape exfoliation method, following which the unique and excellent electrical and mechanical properties of graphene began attracting attention [8,9,10]. Since then, various researchers have actively researched the applications of graphene-based electronic devices. Graphene is a honeycomb-shaped two-dimensional planar carbon allotrope composed of the sp2 bonds of carbon atoms [11]. Graphene is classified as one of the nanostructured carbon allotropes, along with the soccer-ball-like fullerene (C60), columnar carbon nanotubes, and multilayered graphite [12]. Of the four outermost electrons in the carbon atom, three electrons form σ-bonds to produce a hexagonal structure; owing to the long range of π-conjugation of the remaining electron, graphene features excellent physical and electrical properties [13]. Graphene has a 100-fold higher electron mobility than silicon, which is the main raw material for semiconductors, and 100-fold greater electrical conductivity than copper; its tensile strength is more than 200 times that of steel, with excellent elasticity that allows maintenance of the electrical conductivity even if the area is increased or bent by more than 10%. In addition, it has more than twice the thermal conductivity of diamonds [14]. To apply graphene having such excellent properties as a core material in electronic devices, doping is the most effective method as it minimizes physical damage [15,16]. Doping is well known as an effective method to change the electrical properties of a material and is mainly used in technologies involving semiconductors. The basic principle of doping involves adding a material that can donate or remove electrons, thereby changing the electrical properties of the original material. Although the theoretical physical properties of graphene are excellent, in reality, graphene does not have the carrier mobility of theoretical graphene; thus, the surface resistance of the synthesized graphene is relatively high compared to indium tin oxide (ITO) [17]. Thus, it has been noted that electrons and holes are not efficiently transferred in the graphene-based device. To solve this problem, attempts were made to control the work function of graphene and lower its surface resistance by a doping technique mainly used with silicon [18]. In Si-based semiconductor doping technologies, group 3 (boron) or group 5 (nitrogen) ions are used to obtain p- or n-type doped materials or annealed at high temperature with the target material to be doped. However, as graphene is composed of single-layered atoms, it is difficult to apply this method; therefore, new doping technologies must be developed [19,20]. Conventional doping in graphene is similar to that used with silicon technology but proceeds by a different mechanism; this is generally achieved by three main methods, namely substitutional, chemical, and surface charge transfer doping, all of which have distinct characteristics and principles. In particular, doping methods that apply organic molecules or metal particles to the upper surface of graphene by dry deposition to achieve charge transfer have been studied and used in recent years [21]. In general, there are two types of chemical surface modification methods for graphene. One is covalent-bonding technique in which a bond is formed on the surface of graphene and the other is non-covalent-bonding one uses the interaction between graphene and the functional group. First, for the covalent-bonding surface modification method, a functional group is formed at the end of the graphene rather than the surface, and exfoliation is performed by electrostatic repulsion between the functional groups. In this case, as defects are not formed on the surface of graphene, the electrical conductivity of graphene is maintained, and the dispersibility of graphene in a polar organic solvent can be improved due to the electrostatic repulsion. As a result, it becomes easier to manufacture highly conductive composite materials. Second, for the non-covalent-bonding surface modification method, non-covalent functionalization of graphene based on π-π bonding, hydrogen bonding, or interaction among charges reduces defects on the surface of graphene more effectively compared with the covalent functionalization method. Therefore, it has an advantage of not degrading the thermal stability of graphene as well as sustaining the excellent electrical conductivity. On the other hand, there is a drawback in that the dispersibility in a polar organic solvent is reduced compared with the first method. Herein, the characteristics, advantages, and disadvantages of the existing substitution and chemical doping methods are discussed. Furthermore, the basic principles of the organic molecular doping method are examined based on the charge transfer method, with a review of the applications.

### 2.2. Substitutional Doping

**Figure 1:**
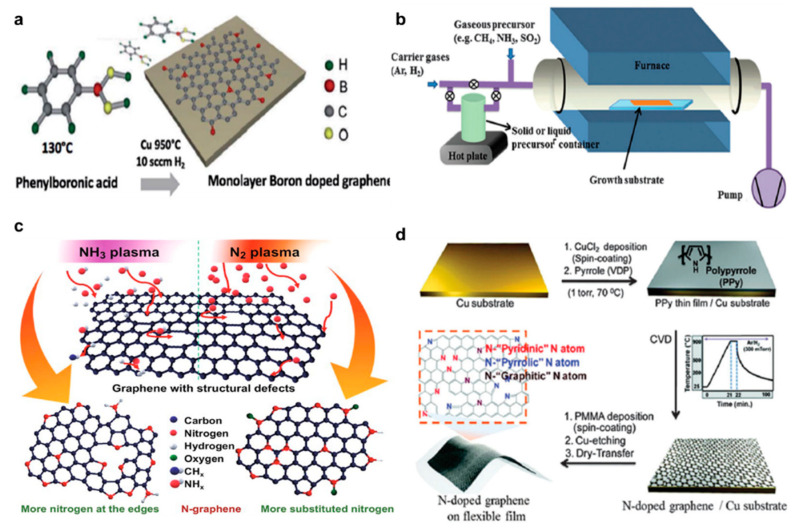
(**a**) Schematic illustration of the chemical vapor deposition (CVD) deposition of B-G on Cu foils by using phenylboronic acid (adapted from [23] with permission from Journal of Materials Chemistry A). (**b**) Experimental setup commonly used for CVD graphene doping (adapted from [25] with permission from Chemical Society Reviews). (**c**) Schematic diagram of the possible mechanism of nitrogen incorporation in CNWs by NH_3_ and N_2_ plasma post-treatment respectively (adapted from [24] with permission from Nano-Micro Letters). (**d**) N-doped graphene derived from polypyrrole [25] (adapted from [25] with permission from Chemical Society Reviews).

Controlling the number of layers is a predominant way for wide-bandgap modification [22]. Hybrid nanosheets demonstrate the energy-bandgap tunability depending on the number of layers. Practical measurement results based on photoluminescence indicates that the energy bandgap is in the negative relation with the increase in number of layers [22]. Being closely related with doping, the major topic of this work, substitutional doping can be also one of the efficient ways of opening the energy bandgap of graphene, as can be proven by previous theoretical works on B, N, and Bi doping in graphene [23]. Also, the effects of substitutional doping on the materials including the change in electronic properties of graphene were studied by the help of ab initio calculations based on density functional theory (DFT) [24]. When graphene is substituted and doped with other hetero-atoms using chemical vapor deposition (CVD), a graphene lattice is formed via a covalent bond with carbon atoms, and this structure is suitable for device fabrication because it can elicit stable and reproducible electrical properties. Therefore, substitution doping is commonly used in silicon technologies [23,24,25,26]. Since the discovery of graphene, many attempts have been made to replace carbon atoms with boron and nitrogen, which are located to the left and right of carbon in the periodic table. Graphene is a zero-bandgap semimetal and does not have semiconductor characteristics, so it is difficult to use as a transistor. Substitution doping has been reported as a useful method to open the band gap of graphene [12]. Here, boron and nitrogen are the best acceptors and donors as their outermost electrons are trivalent or pentavalent and similar in size to those of carbon. Thus, substituting carbon with graphene using boron and nitrogen, experimental results have been reported on opening the valence and conduction bands [12,23]. There are two main methods of graphene substitution doping, of which the first is substitution during graphene synthesis [12]. CVD is the most common method for synthesizing graphene. Boron (HBO_3_, H_2_B_6_) and nitrogen (NH_3_) are added when synthesizing through this method (Figure 1a,b) [12,25]. And graphene substituted with boron and nitrogen is synthesized by flowing methane at the same time. The second substitutional doping method involves heat treatment of graphene at high temperatures or slicing plasma while flowing gases containing the substitution elements over the synthesized graphene [24]. If a gas containing boron and nitrogen is flowed while performing strong heat treatment on graphene oxide, the oxide is reduced and replaced, and the degree of doping can be controlled through the intensity of the plasma and the amount of flowing gas (Figure 1c) [27,28]. The doping efficiency and configuration also critically depend on the precursor (s) such as Melamine, polyaniline and polypyrrole (Figure 1d) [24,29,30,31]. However, it has been noted that this doping method can cause structural defects in graphene, which eventually degrade its conductivity and can pose obstacles when applied to electronic devices [32]. One method of compensating for this shortcoming is chemical doping, which is discussed in the next section.

### 2.3. Chemical Doping

**Figure 2:**
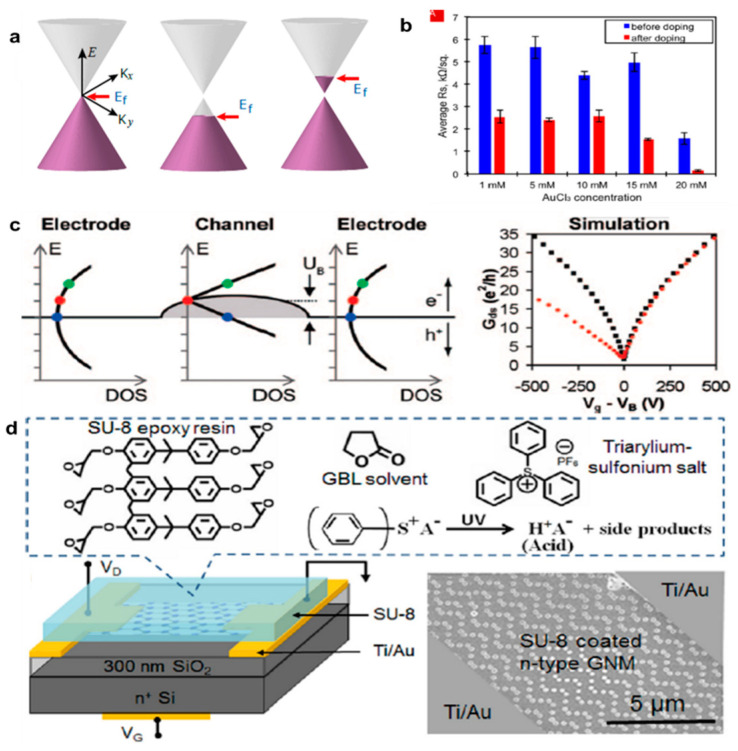
(**a**) Schematic band structures of graphene (adapted from [11] with permission from Insciences Journals). (**b**) Sheet resistance measurements of few-layer graphene (FLG) samples made before and after doping with different AuCl_3_ concentrations (adapted from [34] with permission from Applied Surface Science). (**c**) Non-equilibrium Green’s function (NEGF)-based simulations comparing three types of graphene channel potentials (adapted from [33] with permission from Nano Letters). (**d**) Schematic of an n-type graphene transistor with SU-8 as an n-type dopant and self-encapsulation. Upper diagram described basic chemical compounds of SU-8 and the chemical reaction for generating photo-acid upon UV exposure (adapted from [41] with permission from Applied Physics Letters).

In implementing an electronic device based on a graphene electrode, adjusting the band energy is important to achieve power efficiency and reliability of the device [18]. However, it is difficult to find a material with the desired energy band that also has appropriate conductivity. Therefore, it is more suitable to change the work function by simple surface or chemical treatment. Even in silicon-based device technologies, research on changing the properties using chemical dopants has been underway for more than a decade. Many researchers have developed various doping techniques to effectively control the electrical and optical properties of the carbon-based target material. Doping methods using chemical substances have been widely studied because they do not change the mechanical and chemical properties of the target substance and can easily control the electrical properties [26,33]. Controlling the work functions of carbon materials and improving their electrical properties using chemical doping methods has also been investigated. Doping of graphene using chemicals has been studied continuously through theoretical and experimental methods, and the work function of graphene can be changed to p-type or n-type depending on the chemicals used (Figure 2a) [11]. For example, gold chloride, which is commonly used for doping organic conductors, is the most well-known material for doping graphene; it is a p-type dopant that can increase the work function of graphene and lower its surface resistance (Figure 2b) [34]. Gold chloride dissolves in nitromethane and dissociates into gold trivalent cations and chlorine ions; the reaction by which the gold trivalent cations remove electrons from graphene and reduce to gold nanoparticles occurs spontaneously on the surface of graphene because of the negative Gibbs free energy [16]. Similarly, it has been reported that graphene’s work function and surface resistance can be lowered using metal chlorides with high work functions and negative Gibbs free energies [34]. The work function of graphene is known to be about 4.2–4.4 eV and may increase to 4.7–5.1 eV depending on the concentration of the gold chloride doping solution; further, the surface resistance varies depending on the method of synthesis but may be lowered to 150 Ω/sq after doping [35,36,37]. As stable, complementary dopants of graphene, poly (ethylene imine) and diazonium salts were investigated [33]. The carrier transport properties in graphene devices doped with these molecules exhibit asymmetries in electron–hole conductances; while maintaining the conductance of one carrier, the conductance of the other carrier decreases. As shown in the Figure 2c, the simulation results suggest that the origin of this asymmetry is the imbalanced carrier injection from the graphene electrode caused by misalignment of the neutrality points. In addition, the conductivity of graphene increases rapidly when steam is applied to the surface of graphene with strong acids, such as nitric acid and hydrogenated iodine. Methods to control the work function by changing the electrical properties of graphene using materials such as bis (trifluoromthanesulfonyl) amide (TFSA) and poly (ethylene imine) (PEI) have been published [26,38,39,40]. As an effective electron dopant and a stable encapsulating layer, SU-8 photoresist can be used to achieve highly stable n-type graphene nano-meshes. The chemically stable n-type electro-chemical characteristics of the SU-8-doped graphene were evaluated in air using Raman spectra, electrical transport properties, and electronic band structures [41,42]. 

The SU-8 doping causes minimum damage to the hexagonal carbon lattice structure of the graphene layer and is completely reversible by removal of the uncross-linked SU-8 resist, shown in Figure 2d. In conclusion, the chemical doping method is able to effectively control the fermi-level of graphene with a chemical substance with electronegativity, which has a large gap with carbon, and can be the main technical basis for the charge transfer method described in the following section.

### 2.4. Charge Transfer Method (Molecular Doping)

**Figure 3:**
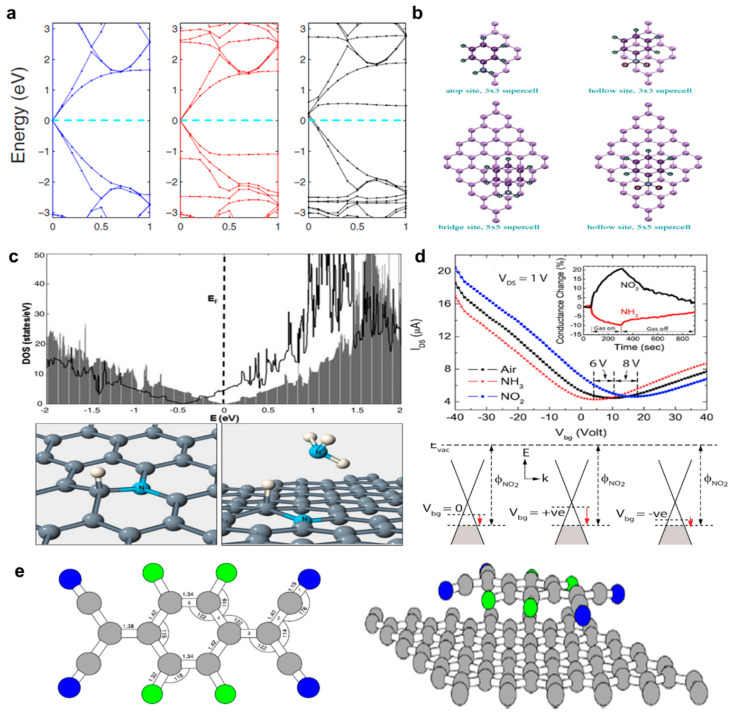
(**a**) Electronic structure of the pristine single-layer graphene, aniline-adsorbed graphene, and nitrobenzene-adsorbed graphene (adapted from [21] with permission from Physical Review B). (**b**) Top view of the lowest-energy configurations of the molecules adsorbed on graphene (adapted from [21] with permission from Physical Review B). (**c**) Electronic density of states (DOS) for graphene with no impurities (shaded area) and with a physiosorbed NH_4_ group (black solid line) (top). N–H complex: H atom trapped at a substitutional N dopant site of graphene (bottom left). A NH_3_ molecule physiosorbed over a N–H complex (C: gray; N: light gray (cyan)); H: white spheres (bottom right) (adapted from [45] with permission from Physical Review B). (**d**) Current-voltage transfer characteristics of aback-gated CVD graphene field-effect transistor (FET) in air and in the presence of NO_2_ and NH_3_. Inset shows the percentage conductance changes of CVD grown multi-layer graphene (MLG) with the flow of 20 ppm NO_2_ and 550 ppm NH_3_ (top). Energy-band diagrams showing shift in Fermi level in back-gated graphene sensor as a result of gate bias and NO_2_ adsorption (bottom) (adapted from [46] with permission from Applied Physics Letters). (**e**) Molecular structure of F4-TCNQ. Bond lengths are in Å and angles in degrees (left). Molecule of F4-TCNQ on top of graphene (right) (adapted from [48] with permission from Journal of Physics: Condensed Matter).

As mentioned above, the Fermi level of graphene could be easily modulated through chemical doping, but more sophisticated technologies are required to create the band gap. The last of the main doping methods for graphene involve surface charge transfer using an organic molecular polymer material. Doping can be performed by exchange of electrons between semiconductor and dopants adsorbed on the semiconductor surface in case of surface transfer doping technique. For this reason, this technique is also called as adsorbate-induced doping. In general, the surface transfer doping does not ruin the atomic arrangement of graphene and has high process repeatability [26]. As noted previously, doped graphene for various electrical properties could be obtained through direct synthesis and post treatments. Among those post treatment methods, wet doping methods can be performed using acid, metal chloride treatment, and organic molecular material coatings on the graphene surface using dip, spin coating, etc. [16]. When a molecule capable of causing charge transfer is applied to the surface of graphene or a thin doping film is deposited, charge transfer occurs spontaneously owing to the difference in electronegativity from the carbon constituting graphene. This mechanism has been widely introduced as an effective method for doping graphene. Theoretically, as shown in Figure 3a,b, DFT was employed in order to investigate changes in the electronic structure and vibrational property of graphene when aromatic molecules such as aniline and nitrobenzene are adsorbed [21]. The simulation results explicitly reveal the importance of dynamic corrections to phonon frequency of molecular-doped graphene, which have been previously proven to be crucial for electrochemical and substitutional doping with either B or N. These findings clearly contrast between the electrochemical and molecular doping methods of graphene and have merits such as controllability of the dopant, scalability, and ease of use over the direct synthesis methods. Using this method, it is possible to functionalize graphene by effectively controlling its band gap, thereby increasing applicability to various types of electronic devices. To implement the organic molecular doping method described herein, methods of doping at room temperature using chemicals having high or low electronegativities compared to carbon have been attempted [43,44]. Depending on whether number of electrons is insufficiently small or large enough on the graphene surface, the type of graphene is determined to be either p-type or n-type by accepting or repelling the electrons from the surface. The charge transfer takes place by the combinational determination by the graphene Fermi level, the highest occupied molecular orbital (HOMO), and the lowest unoccupied molecular orbital (LUMO) levels of the species adsorbed on the graphene surface. If the HOMO level of the adsorbent on graphene is higher than the graphene Fermi level, the adsorbent becomes a donor that transfers electrons to graphene. Conversely, when the LUMO of the adsorbent is higher than the Fermi level of graphene, charge transfer is allowed to take place from the graphene layer to the adsorbent which acts as the acceptor. Previous literature addresses that ammonium group (NH_4_) can be an ideal candidate for molecular doping [45]. The group meets the requirements, in the quantitative manner, for stable physisorption and efficient doping in graphene. The ammonium groups usually demonstrate relatively large physisorption energies more than 1 eV and effectively transform graphene into an n-type material. Also, the ammonium radicals show relatively large binding energies around 1.55 eV, compared with other small molecules, when they are physiosorbed on the graphene surface (Figure 3c). Tunability of molecular doping in an electrical matter was investigated in a back-gated field-effect transistors by Singh et al. (Figure 3d) [46]. The amount of charge transfer doping showed a monotonic decrease for one of the representative p-type dopants, gaseous dopant NO_2_, when the back-gate voltage increased in the negative direction. On the other hand, it showed a monotonic increase when n-type gaseous dopant NH_3_ was employed. The series of results strongly support that utilization of the principle of adsorption-driven doping technique can be expected for controlling the sensitivity and selectivity in molecular detector applications. For graphene, tetrafluoro-tetracyanoquinodimethane (F4-TCNQ) and fluoropolymer (CYTOP) are well known as adsorbents that cause active surface charges [44,47,48]. F4-TCNQ is a very strong electronic acceptor, and recently, the results of increasing the work function to 5.24 eV in graphene doping using this acceptor was reported [44]. The transfer of charge from F4-TCNQ to graphene is caused by the cyano functional groups, which are part of the structure of F4-TCNQ. This functional group is a structure in which electrons are insufficient and serve to strongly pull electrons from graphene. Owing to this, graphene provides electrons to F4-TCNQ, thereby forming a depletion layer and achieving p-type doping [47,48]. In the case of CYTOP, it replaces PMMA, which is mainly used to transfer graphene and has advantages where graphene doping can proceed simultaneously with transfer. This polymer support layer is a type of fluorine compound and can be easily structurally bonded to the carbon of graphene to cause p-type doping by bonding through a simple annealing process. Owing to the insulating characteristics of CYTOP, charge transfer cannot naturally occur between CYTOP and graphene. However, it has been reported that owing to the differences in electrostatic attraction, a dipole moment between graphene and CYTOP causes p-type doping of graphene [49,50]. That is, when a molecule capable of causing charge transfer is applied on graphene or a structure formed through deposition, charge transfer may occur spontaneously by the difference in electronegativity with carbon constituting graphene. Based on these advantages, organic molecular doping is expected to be the most effective method for doping graphene without an additional annealing process.

## 3. TMD Doping

### 3.1. Background

TMD doping techniques are classified in two ways: (i) atom-substitutional doping [51,52,53,54,55,56,57,58,59] and (ii) molecular doping. Substitutional doping replaces transition-metal (or chalcogen) atoms with dopants and has the merit of not damaging the crystal structure. However, control of the relative amounts of the different atoms is still challenging. As a result, accurate doping control for TMD materials doped with substitutional doping techniques has not yet been reported. Meanwhile, in molecular doping, the doping technique is divided into two mechanisms: (i) charge transfer from the dopant molecules to the TMDs and (ii) dipole effects of dopant molecules. TMDs are doped by transferring carriers directly from the dopant molecule to the TMD by the potential difference of the dopant molecule and the TMD. The dipole effect of the molecular dopant also provides electrical doping of TMDs. A typical feature of TMDs is the Schottky barrier (SB) of TMD–metal junctions. The SB prevents charge injection from the contact electrode to TMDs, which limits the electrical performance, including the effective charge carrier mobility and subthreshold swing. Additional dipole effects in the molecular dopant modify the energy structure of the TMD; hence, the SB width at the TMD and metal interface can be modulated by the dipole moment of the molecular dopant. In particular, in the case of SAMs, forming a junction with the TMD surface and the alignment structure of the monolayer has an effective dipole effect. Furthermore, SAMs adjust the dipole direction (i.e., negative or positive dipole) and intensity depending on their functional group. However, both (i) atom-substitutional doping and (ii) molecular doping techniques have several limitations, including environmental and operational stability of the doping effect, off-current increase, and difficulty of accurate doping control. This section describes the features, advantages and disadvantages of recently reported substitution and molecular doping methods on TMDs. In addition, the fundamental principles of the organic molecular doping process are described, and its application to photodetectors, complementary circuits, and neuromorphic devices are reviewed.

### 3.2. Substitutional Doping

**Figure 4:**
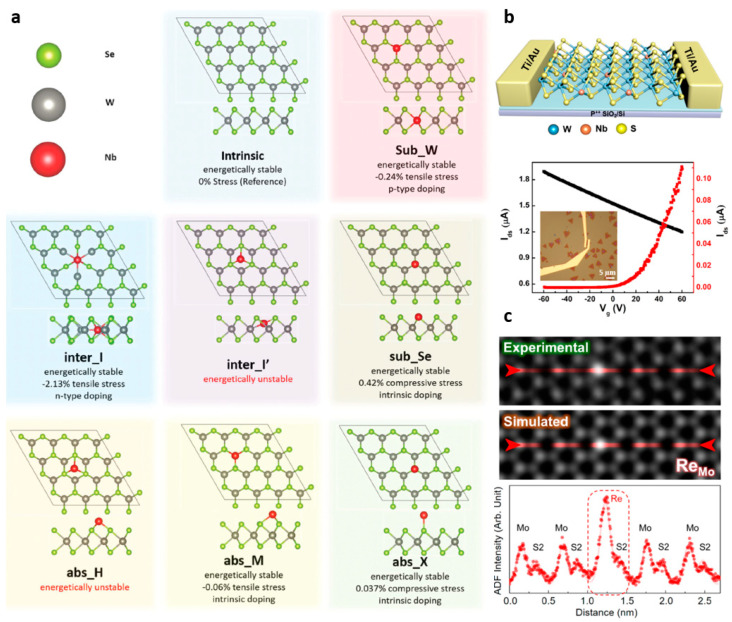
(**a**) Nb-doped WSe_2_ monolayer for DFT calculations (adapted from [51] with permission from Royal Society of Chemistry). (**b**) Schematic of monolayer Nb_x_W_1−x_S_2_ and transfer characteristics of the monolayer (black line is Nb_x_W_1−x_S_2_, red line is WS_2_) (adapted from [55] with permission from ACS publications). (**c**) Experimental and simulated STEM images and line scans of Re and Mo dopants; the dots in the line scans represent experimental results, whereas the solid lines represent simulated results (adapted from [59] with permission from ACS publications).

Much efforts have been made to dope TMDs to precisely control their electrical properties, and intrinsic semiconductors are becoming more important. One approach is to produce doped TMDs by substitution of (i) transition metal [51,52,53,54,55,56,57,58,59] or (ii) chalcogen atoms [60,61,62,63,64,65,66] within the TMD lattice. In TMD composed of transition metal (M) interposed between two chalcogen (X) atomic layers, substitutional doping of TMD is realized through replacement of the transition metal atom (e.g., Mo or W) or chalcogen atom (e.g., S, Se, or Te) with another atom. This doping approach forms a new chemical bond, which provides higher doping effect stability when compared to charge transfer doping. Using the in-situ synthesis doping process of the acceptor Niobium (Nb), substitutionally Nb-doped WSe_2_ can be fabricated (Figure 4a) [51,55,56,58]. The doping process substituted by Nb enables reducing the contact resistance for hole charge carriers, and as a result, high-performance p-type operation as high as 116 cm^2^V^−1^s^−1^ with an on/off ratio of 10^6^ was obtained (Figure 4b) [51]. Besides, there are successful cases of substitutional doping based on various transition metal atoms, such as iron (Fe), rhenium (Re), and vanadium (V), achieving Fe-doped WS_2_, Re-doped MoS_2_, and in-plane heterostructures, such as V-doped W_x_Mo_1−x_S_2_−Mo_x_W_1−x_S_2_ by liquid-phase precursor-assisted synthesis (Figure 4c) [59]. 

**Figure 5:**
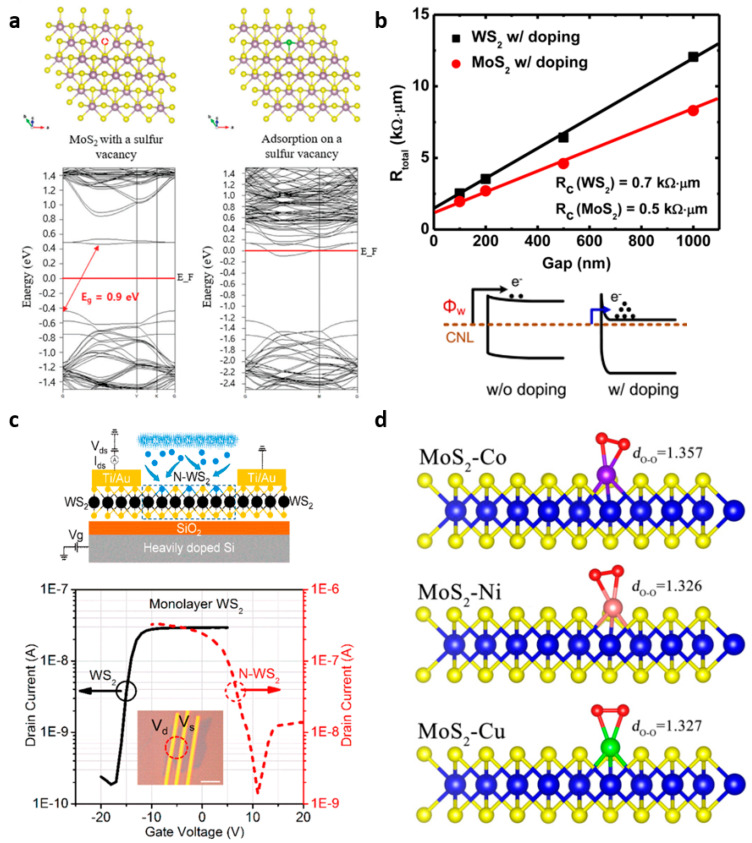
(**a**) Change in band structure for adsorption of chlorine atom on S vacancy of MoS_2_ (adapted from [64] with permission from Royal Society of Chemistry). (**b**) Transfer length method (TLM) resistances of Cl-doped WS_2_ and MoS_2_ and schematic band diagram of metal-transition metal dichalcogenide (TMD) contacts with and without chloride doping (adapted from [62] with permission from ACS publications). (**c**) Transfer characteristics at *V*_DS_ = 1 V for pristine WS_2_ and N-WS_2_ transistors (adapted from [63] with permission from ACS publications). (**d**) Geometric structure and charge density difference for intermediate OOH adsorption on the hydrogenated MoS_2_-TM (Co, Ni and Cu) system; the gray, red, violet, pink, green, blue, and yellow spheres represent H, O, Co, Ni, Cu, Mo and S atoms, respectively (adapted from [61] with permission from Elsevier).

Recently, the n-doping effect in WS_2_ was reported for Sn-substituted Sn_x_-W_1-x_-S_2_ through post-growth substitution [57]. In addition to transition metal substitution, the chalcogen atom can be substituted with chlorine (Cl) [62,64], nitrogen (N) [60,63], or oxygen (O) [61,65]. Cl substitution doping by remote inductively coupled plasma (ICP) allowed MoS_2_ to be n-doped with a Fermi level shift close to the conduction band (Figure 5a) [64]. Electron irradiation beam (e-beam) causes atom sputtering to create a sulfur vacancy, which enables post-synthesis doping [66]. Another technique to produce chalcogen-atom-substituted doping is dipping in 1,2-dichloroethane (DCE) solution, resulting in a reduction of the SB width with electrical n-doping effects (Figure 5b) [62]. The Cl-doping enables the contact resistances of WS_2_ and MoS_2_ to decrease to 0.7 kΩ·μm and 0.5 kΩ·μm, respectively. Meanwhile, p-doping effects can be obtained by substitution with N atoms on chalcogen vacancies. Atomic nitrogen treatment by plasma-confinement plate induced p-type behaviors of nitrogen-doped WS_2_ from the baseline n-type WS_2_, which enabled complementary device operation of WS_2_ (Figure 5c) [63]. In addition to atomic substitutions, hydrogenated MoS_2_-TM (Co, Ni and Cu) can be obtained through the adsorbed O_2_ with the formation of OOH radical (Figure 5d) [61]. Thus, it is important to form accurately controlled vacancies in TMDs where the dopants are implanted. 

### 3.3. Molecular Doping

**Figure 6:**
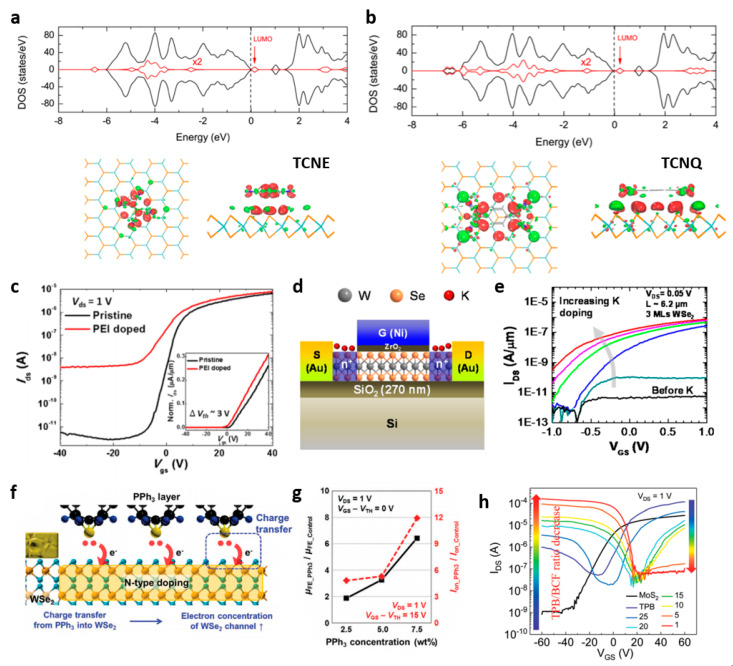
(**a**,**b**) Charge transfer between molecular donors (tetracyanoethylene (TCNE) and tetracyanoquinodimethane (TCNQ)) and MoS_2_ with sulfur vacancies. Total density of states (DOS) (black) and local density of state (LDOS) of the molecule (red) (adapted from [72] with permission from ACS publications). (**c**) Transfer characteristics of pristine and poly (ethylene imine) (PEI)-doped multilayer MoS_2_ transistors (adapted from [85] with permission from John Wiley and Sons). (**d**) Schematic of a top-gated few-layer WSe_2_ transistor with chemically n-doped source/drain contacts by K exposure [75]. (**e**) Transfer characteristics of a 3-layer WSe_2_ device (L∼6.2 μm) as a function of K exposure time (adapted from [75] with permission from ACS publications). (**f**) Descriptive diagram for n-doping mechanism of PPh_3_ at the PPh_3_/WSe_2_ interface (adapted from [84] with permission from John Wiley and Sons). (**g**) Extracted field-effect mobility ratio (μ_FE_ ratio = μ_FE_PPh3_/μ_FE_Control_) at *V*_GS_ = *V*_TH_ (adapted from [84] with permission from John Wiley and Sons). (**h**) Transfer characteristics of MoS_2_ device on a semi-log scale with gradually decreasing ratios of TPB/BCF (*V*_DS_ = 0.1 V) (adapted from [83] with permission from Royal Society of Chemistry).

Compared to substitutional doping techniques, molecular doping has several advantages: low-cost processing and large area coverage. By simply coating or depositing a film that provides a doping effect, the electrical properties of the TMDs, such as effective mobility, threshold voltage, and subthreshold swing, can be adjusted. This approach enables forming a heterostructure of the doping film and the TMD, in which the electrical or optical characteristics of the TMDs can be enhanced by (i) charge transfer from the dopant molecules to TMDs [62,67,68,69,70,71,72,73,74,75,76,77,78,79,80,81,82,83,84,85,86,87,88,89,90,91,92] or (ii) dipole effects of the dopant molecules of the doping film [4,5,6,7,93,94,95]. The energy band structure of MoS_2_ could be controlled through molecular doping from the deposited tetrathiafulvalene and dimethyl-phenylenediamine molecules as donors and tetracyanoethylene (TCNE) and tetracyanoquinodimethane (TCNQ) as acceptors (Figure 6a,b) [72,88]. Solution-coating of polyethyleneimine (PEI) showed effective doping on device characteristics by reducing the contact resistance; accordingly, effective mobility was improved by 1.6 times (Figure 6c) [68,85]. However, (i) charge transfer effects from dopant molecules could be easily degenerated as this doping approach is fundamentally a reaction by physical adsorption rather than doping by chemical bonding. For example, in MoS_2_ and WSe_2_, the charge transfer doping with enhanced electrical properties was achieved with potassium [75], but the doping effect was not stable in air (Figure 6d). Thus, air-stability in molecular doping has been considered as an important developmental aspect. Benzyl viologen (BV) provided air-stable n-doping effects on MoS_2_, and as a result, a subthreshold swing of 77 mV/decade and high sheet electron density of 1.2 × 10^13^ cm^−2^ were obtained in ambient air. Triphenylphosphine (PPh_3_) was used as a dopant with controllable non-degenerate doping, which provided robust doping of MoS_2_ for 14 days in ambient air (Figure 6e,f) [2,88]. By controlling the ratio of the two dopants: tris(pentafluorophenyl)borane (BCF), containing electron-withdrawing fluorine, and tritolylborane (TTB), containing electron donor methyl groups, the operation behaviors of TMDs were controlled from ambipolar to unipolar switching characteristics (Figure 6g) [2,88]. 

**Figure 7:**
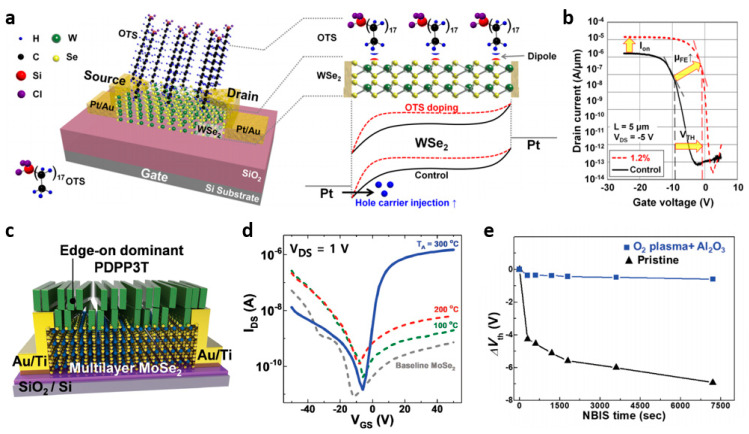
(**a**) Schematic of back-gated transistor fabricated on octadecyltrichlorosilane (OTS)-doped WSe_2_ and energy band diagrams of Pt-WSe_2_-Pt junctions (adapted from [79] with permission from ACS publications). (**b**) Transfer characteristics of transistors fabricated on (black) undoped and (red) 1.2% OTS-doped WSe_2_ devices (adapted from [79] with permission from ACS publications). (**c**) Schematic of PDPP3T-doped MoSe_2_ transistor (adapted from [93] with permission from John Wiley and Sons). (**d**) Transfer characteristics of PDPP3T-doped MoSe_2_ as a function of annealing temperature (adapted from [93] with permission from John Wiley and Sons). (**e**) Comparison of shifts in *V*_TH_ derived from NBIS test results (adapted from [91] with permission from John Wiley and Sons).

The doping effect by (ii) dipole of dopant molecules showed non-degenerated effects. The SAM treatment of octadecyltrichlorosilane (OTS) caused a positive dipole moment at the WSe_2_-OTS interface, enabling the p-doping phenomenon (Figure 7a,b) [4,6]. On the other hand, the SAM treatment of (3-aminopropyl) trimethoxysilane (APTMS) caused a negative dipole moment, turning out the n-doping of rhenium diselenide (ReSe_2_) [90,96]. Because the SAM materials are aligned to form a junction on the surface of the TMDs, the interface dipole is effectively provided [97,98,99,100,101]. Oxide dopantsof transition metal oxides (TMO) also provide non-degenerated doping effects on TMDs [71]. TiO_2_ and MoO_3_ form interface dipole, enabling n- and p-doping of MoS_2_, respectively, non-degenerately. Poly-(diketopyrrolopyrrol-eterthiophene) (PDPP3T) as a polymer semiconductor exhibited a high doping effect on multilayer MoSe_2_ and MoS_2_, leading to significant improvements in the on-current (≈×2000 higher) (Figure 7c,d) [93,94]. Polymer chains in PDPP3T assist alignment with directionality in junction structure with TMDs, providing effective charge transfer and dipole effects. To provide high-stable doping effects, a two-step functionalization doping scheme was developed. The two-step n-doping process based on oxygen plasma treatment and Al_2_O_3_ deposition allowed MoSe_2_ transistors to operate as unipolar n-doped behavior, and the negative bias illumination stress (nbis) test for 7200 s and the environmental stability test for 21 days showed unchanged stable doping effect (Figure 7e) [91]. Although the molecular doping techniques have been intensively studied for control of the TMD devices, uniformity and controllability still remain as challenging issues. 

### 3.4. Applications

**Figure 8:**
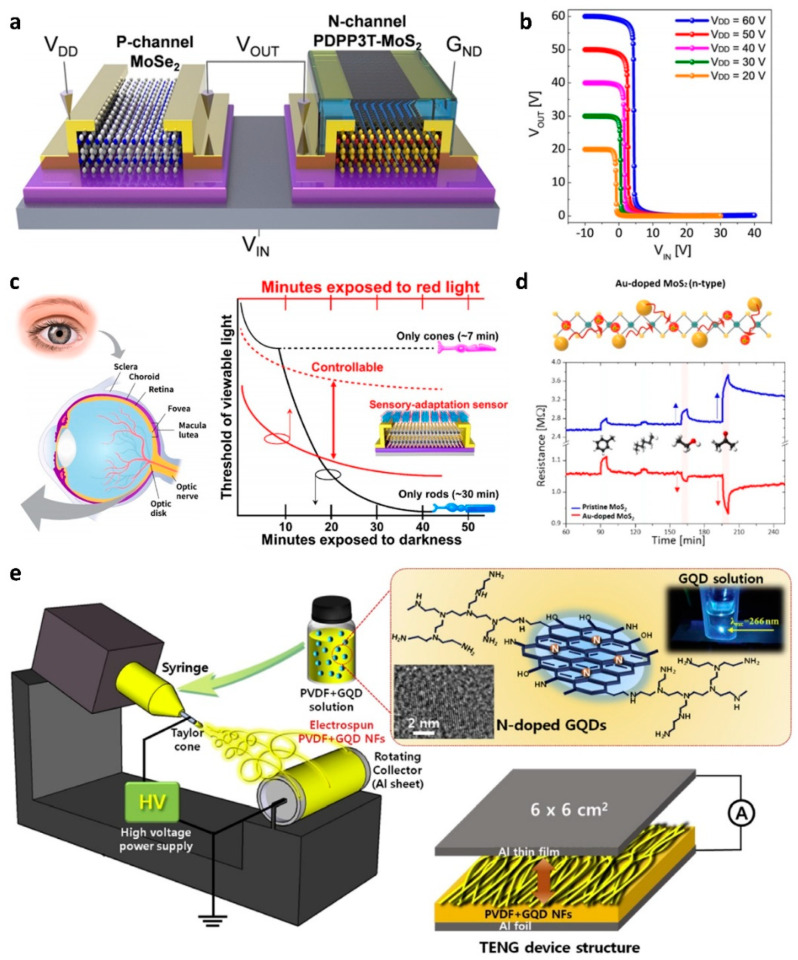
(**a**) Schematic of complementary metal-oxide-semiconductor (CMOS) inverter using doped-MoS_2_ and MoSe_2_ transistors (adapted from [94] with permission from ACS publications). (**b**) Transfer curves of CMOS inverter using doped-MoS_2_ and MoSe_2_ transistors (adapted from [94] with permission from ACS publications). (**c**) Sensory adaptation of the eye and CsPb(Br_0.5_I_0.5_)_3_−MoS_2_ phototransistor (adapted from [89] with permission from ACS publications). (**d**) Real-time resistances of pristine MoS_2_ and Au-doped MoS_2_ sensors exposed to various VOCs (adapted from [109] with permission from ACS publications). (**e**) Triboelectric nanogenerator using n-doped graphene quantum dots and polyvinylidenefluoride (PVDF) (adapted from [110] with permission from Elsevier).

The electrical properties can be controlled through TMD doping, and various applications of photodetectors [84,86], complementary circuits [73,74], and neuromorphic devices [89] have been actively demonstrated. As the doping techniques modulate the energy band of TMDs, not only electrical properties but also optical properties can be improved through doping [4,7,84,86,89,90,94,96]. Higher photoresponsivity and temporal photoresponse performance could be achieved by the surface charge transfer doping. Another application is to build complementary circuits by selective doping of TMDs [73,74,80,81,94]. Pristine TMD devices suffer from ambipolar characteristics, normally-on operation due to *V*_TH_ shift, and contact resistance, which limit implementation to complementary circuits. High DC voltage gain of the complementary inverter as high as ~170 V/V was obtained by p-type MoSe_2_ transistor and the PDPP3T-doped n-type MoS_2_ transistor (Figure 8a,b) [94]. Recently emerging applications are neuromorphic devices such as synaptic transistors and artificial neurons [102]. SB barrier modulation by defect doping and 1T′ (metal) phase transition by lithium-ion intercalation enabled MoS_2_ to operate as a synaptic memristor behavior. Neuromorphic phototransistors were demonstrated by using charge transfer effects in perovskite and MoS_2_ hybrid structure, which emulated human sensory adaptation (Figure 8c) [89]. Furthermore, the improvement of sensing properties can be achieved by the doping methods in 2D materials [102,103,104,105,106,107,108,109]. As an example, Au nanoparticles on MoS_2_ induced n-doping effects, which significantly enhanced chemical sensing properties with respect to various volatile organic compounds (Figure 8d) [109]. Another emerging application based on the doping process in 2D materials is found in nanomechanics [110,111,112,113]. To make high performance nanogenerators with larger output power, formation of surface charges or control of work functions of 2D materials is crucial. Graphene quantum dots were n-doped with nitrogen, providing high photoluminescence (PL) quantum efficiency above than 90% [110]. Also, negatively-charged graphene surface had an ion-dipole interaction with the positive charged in the polyvinylidenefluoride (PVDF), which resulted in enhanced triboelectric nanogenerator (Figure 8e). Furthermore, AuCl_3_-doped crumpled graphene enabled the triboelectric nanogenerator to operate with higher performance of output voltage and current = 80.6 V and 11.9 μA/cm^2^ at the doping level of 1.2 mg/mL [111]. Because doping technique can substantially control the electronic structure of a TMD, various doping processes can be applied according to the requirements for specific device application (Table 1).

## 4. Conclusions and Outlook

In this review, we have provided an overview of important recent researches on doping strategies covering substitutional, chemical and molecular methods for graphene and advanced two-dimensional semiconducting materials. Owing to the atomically thin nature and dangling-bond free surface, two-dimensional semiconducting materials are regarded as a strong candidate for the viable channel materials that can prevent short-channel effects. To further expand the potential applications of these two-dimensional semiconductor materials, technologies capable of precisely controlling the electrical properties of the material are essential. Recently, there is a continuous demand for the development of doping techniques for these two-dimensional semiconducting materials that can improve device performance by precisely controlling the carrier concentration. Doping has been traditionally used to effectively change the electrical and electronic properties of materials through relatively simple processes. To change the electrical properties, substances that can donate or remove electrons are added. Doping of atomically thin two-dimensional semiconductor materials is similar to that used for silicon but has a slightly different mechanism. When it comes to doping techniques accompanying doping-induced bandgap modification, the strain effects should be considered doubtlessly. One of the most recent articles reveals that bandgap engineering can be expected in the TMD semiconductor materials [22], which can be substantially carried out in doping process simultaneously in some cases. At the same time, the interfacial status should be recursively studied in this doping-induced bandgap modulation scenario because the increase in instability of the interface states results in the degradation of carrier mobilities and unwanted memory effects which threaten ideal transistor characteristics. While more advanced doping techniques with higher reliability and process repeatability are developed, these two practical concerns can be more delved into than ever toward integrated circuit and system-level applications of the graphene and TMD semiconductor materials. This review presented an overview of recent advanced doping techniques covering substitutional, chemical and molecular methods of graphene and TMDs, which are the representative 2D semiconductor materials. 

## Figures and Tables

**Table 1:** Comparison among TMD doping techniques with respect to doping process temperature, features, and applications.

Category	TMD	Dopant	Doping Process Temperature (°C)	Features	Device and Application	Ref.
Substitutional doping	WS_2_	Nb	800–830 (doping in synthesis)	Supply of Nb/ Lattice Strain	Transistor	[55,56]
Substitutional doping	MoS_2_	Nb/Re	750 (doping in synthesis)	Supply of Nb and Re	Transistor	[58]
Substitutional doping	MoS_2_/ WS_2_	Fe, Re, and V	700–800 (doping in synthesis)	Liquid-phase precursor-assist	N/A	[59]
Substitutional doping	MoS_2_	N	~200 (doping in synthesis)	Hydrothermal method	Supercapacitor	[60]
Molecular doping	MoS_2_	Benzyl viologen	Room temperature	Air-stable doping	Transistor	[2]
Molecular doping	MoS_2_/ WS_2_	Cl	Room temperature	Using 1, 2 dichloroethane (DCE)	Transistor	[62]
Molecular doping	MoS_2_	Cl	N/A	Cl in remote plasma	Transistor	[64]
Molecular doping	MoS_2_	Oleylamine	300	Contact resistance analysis	Transistor	[69]
Molecular doping	WSe_2_	Triphenylphosphine	N/A	Charge transfer doping	Transistor/ photodetector	[84]
Molecular doping	MoS_2_	Polyethylenimine	Room temperature	Charge injection	Transistor/ photodetector	[85]
Molecular doping	MoS_2_	CsPb(Br1–xIx)3 perovskite	100	Phase segregation effect	Sensory adaptive photodetector	[89]
Molecular doping	MoSe_2_, MoS_2_	PDPP3T	300	Schottky barrier modulation	Photodetector/ CMOS inverter	[93,94]
Molecular doping	MoS_2_	Au nanoparticle	Room temperature	Charge transfer doping	VOCs sensor	[109]

## References

[1] Rai A., Valsaraj A., Movva H.C., Roy A., Ghosh R., Sonde S., Kang S., Chang J., Trivedi T., Dey R. (2015). Air stable doping and intrinsic mobility enhancement in monolayer molybdenum disulfide by amorphous titanium suboxide encapsulation. Nano Lett..

[2] Kiriya D., Tosun M., Zhao P., Kang J.S., Javey A. (2014). Air-stable surface charge transfer doping of MoS_2_ by benzyl viologen. J. Am. Chem. Soc..

[3] McDonnell S., Addou R., Buie C., Wallace R.M., Hinkle C.L. (2014). Defect-dominated doping and contact resistance in MoS_2_. ACS Nano.

[4] Kang D.H., Kim M.S., Shim J., Jeon J., Park H.Y., Jung W.S., Yu H.Y., Pang C.H., Lee S., Park J.H. (2015). High-performance transition metal dichalcogenide photodetectors enhanced by self-assembled monolayer doping. Adv. Funct. Mater..

[5] Li Y., Xu C.-Y., Hu P., Zhen L. (2013). Carrier control of MoS_2_ nanoflakes by functional self-assembled monolayers. ACS Nano.

[6] Lee W.H., Park Y.D. (2018). Tuning Electrical Properties of 2D Materials by Self-Assembled Monolayers. Adv. Mater. Interfaces.

[7] Kang D.-H., Jeon M.H., Jang S.K., Choi W.-Y., Kim K.N., Kim J., Lee S., Yeom G.Y., Park J.-H. (2017). Self-assembled layer (SAL)-based doping on black phosphorus (BP) transistor and photodetector. ACS Photonics.

[8] Geim A.K. (2009). Graphene: Status and prospects. Science.

[9] Miao F., Wijeratne S., Zhang Y., Coskun U., Bao W., Lau C. (2007). Phase-coherent transport in graphene quantum billiards. Science.

[10] Morozov S.V., Novoselov K.S., Katsnelson M., Schedin F., Ponomarenko L., Jiang D., Geim A.K. (2006). Strong suppression of weak localization in graphene. Phys. Rev. Lett..

[11] Guo B., Fang L., Zhang B., Gong J.R. (2011). Graphene doping: A review. Sci. J..

[12] Lee H., Paeng K., Kim I.S. (2018). A review of doping modulation in graphene. Synth. Met..

[13] Shen X., Liu Y., Pang Y., Yao W. (2013). Conjugation of graphene on Au surface by π–π interaction and click chemistry. Electrochem. Commun..

[14] Kumar C.V., Pattammattel A. (2017). Introduction to Graphene: Chemical and Biochemical Applications.

[15] Brownlie L., Shapter J. (2018). Advances in carbon nanotube n-type doping: Methods, analysis and applications. Carbon.

[16] Oh J.S., Kim K.N., Yeom G.Y. (2014). Graphene doping methods and device applications. J. Nanosci. Nanotechnol..

[17] Hemasiri B.W., Kim J.-K., Lee J.-M. (2017). Fabrication of highly conductive graphene/ITO transparent bi-film through CVD and organic additives-free sol-gel techniques. Sci. Rep..

[18] Xue Y., Wu B., Bao Q., Liu Y. (2014). Controllable synthesis of doped graphene and its applications. Small.

[19] Warner J.H., Schaffel F., Rummeli M., Bachmatiuk A. (2012). Graphene: Fundamentals and Emergent Applications.

[20] Sood A.K., Lund I., Puri Y.R., Efstathiadis H., Haldar P., Dhar N.K., Lewis J., Dubey M., Zakar E., Wijewarnasuriya P. (2015). Review of graphene technology and its applications for electronic devices. Graphene New Trends Dev..

[21] Saha S.K., Chandrakanth R.C., Krishnamurthy H., Waghmare U. (2009). Mechanisms of molecular doping of graphene: A first-principles study. Phys. Rev. B.

[22] Chaves A., Azadani A.G., Alsalman H., da Costa D.R., Frisenda R., Chaves A.J., Song S.H., Kim Y.D., He D., Zhou J. (2020). Bandgap engineering of two-dimensional semiconductor materials. NPJ 2D Mater. Appl..

[23] Agnoli S., Favaro M. (2016). Doping graphene with boron: A review of synthesis methods, physicochemical characterization, and emerging applications. J. Mater. Chem. A.

[24] Santhosh N.M., Filipič G., Kovacevic E., Jagodar A., Berndt J., Strunskus T., Kondo H., Hori M., Tatarova E., Cvelbar U. (2020). N-graphene nanowalls via plasma nitrogen incorporation and substitution: The experimental evidence. Nano-Micro Lett..

[25] Wang X., Sun G., Routh P., Kim D.-H., Huang W., Chen P. (2014). Heteroatom-doped graphene materials: Syntheses, properties and applications. Chem. Soc. Rev..

[26] Liu H., Liu Y., Zhu D. (2011). Chemical doping of graphene. J. Mater. Chem..

[27] Manojkumar P., Krishna N.G., Mangamma G., Albert S. (2019). Understanding the structural and chemical changes in vertical graphene nanowalls upon plasma nitrogen ion implantation. Phys. Chem. Chem. Phys..

[28] Hojati-Talemi P., Simon G.P. (2011). Field emission study of graphene nanowalls prepared by microwave-plasma method. Carbon.

[29] Lee V., Dennis R.V., Schultz B.J., Jaye C., Fischer D.A., Banerjee S. (2012). Soft X-ray absorption spectroscopy studies of the electronic structure recovery of graphene oxide upon chemical defunctionalization. J. Phys. Chem. C.

[30] Girard-Lauriault P.-L., Illgen R., Ruiz J.-C., Wertheimer M.R., Unger W.E. (2012). Surface functionalization of graphite and carbon nanotubes by vacuum-ultraviolet photochemical reactions. Appl. Surf. Sci..

[31] Rybin M., Pereyaslavtsev A., Vasilieva T., Myasnikov V., Sokolov I., Pavlova A., Obraztsova E., Khomich A., Ralchenko V., Obraztsova E. (2016). Efficient nitrogen doping of graphene by plasma treatment. Carbon.

[32] Banhart F., Kotakoski J., Krasheninnikov A.V. (2011). Structural defects in graphene. ACS Nano.

[33] Farmer D.B., Golizadeh-Mojarad R., Perebeinos V., Lin Y.-M., Tulevski G.S., Tsang J.C., Avouris P. (2009). Chemical doping and electron−hole conduction asymmetry in graphene devices. Nano Lett..

[34] Abdullah-Al-Galib M., Hou B., Shahriad T., Zivanovic S., Radadia A.D. (2016). Stability of few layer graphene films doped with gold (III) chloride. Appl. Surf. Sci..

[35] Shin H.-J., Choi W.M., Choi D., Han G.H., Yoon S.-M., Park H.-K., Kim S.-W., Jin Y.W., Lee S.Y., Kim J.M. (2010). Control of electronic structure of graphene by various dopants and their effects on a nanogenerator. J. Am. Chem. Soc..

[36] Gunes F., Shin H.-J., Biswas C., Han G.H., Kim E.S., Chae S.J., Choi J.-Y., Lee Y.H. (2010). Layer-by-layer doping of few-layer graphene film. ACS Nano.

[37] Kwon K.C., Choi K.S., Kim S.Y. (2012). Increased work function in few-layer graphene sheets via metal chloride doping. Adv. Funct. Mater..

[38] Kwon K.C., Kim B.J., Lee J.-L., Kim S.Y. (2013). Effect of anions in Au complexes on doping and degradation of graphene. J. Mater. Chem. C.

[39] Jeong H.K., Kim K.-J., Kim S.M., Lee Y.H. (2010). Modification of the electronic structures of graphene by viologen. Chem. Phys. Lett..

[40] Krishnamurthy S., Lightcap I.V., Kamat P.V. (2011). Electron transfer between methyl viologen radicals and graphene oxide: Reduction, electron storage and discharge. J. Photochem. Photobiol. A Chem..

[41] Al-Mumen H., Dong L., Li W. (2013). SU-8 doped and encapsulated n-type graphene nanomesh with high air stability. Appl. Phys. Lett..

[42] Al-Mumen H. (2015). Characterisation of SU-8 n-doping carbon nanotube-based electronic devices. Micro Nano Lett..

[43] Wehling T., Novoselov K., Morozov S., Vdovin E., Katsnelson M., Geim A., Lichtenstein A. (2008). Molecular doping of graphene. Nano Lett..

[44] Chen W., Qi D., Gao X., Wee A.T.S. (2009). Surface transfer doping of semiconductors. Prog. Surf. Sci..

[45] Tsetseris L., Pantelides S.T. (2012). Molecular doping of graphene with ammonium groups. Phys. Rev. B.

[46] Singh A., Uddin M., Tolson J., Maire-Afeli H., Sbrockey N., Tompa G., Spencer M., Vogt T., Sudarshan T., Koley G. (2013). Electrically tunable molecular doping of graphene. Appl. Phys. Lett..

[47] Park J., Jo S.B., Yu Y.J., Kim Y., Yang J.W., Lee W.H., Kim H.H., Hong B.H., Kim P., Cho K. (2012). Single-gate bandgap opening of bilayer graphene by dual molecular doping. Adv. Mater..

[48] Pinto H., Jones R., Goss J., Briddon P. (2009). p-type doping of graphene with F4-TCNQ. J. Phys. Condens. Matter.

[49] Ishikawa R., Bando M., Morimoto Y., Sandhu A. (2011). Doping graphene films via chemically mediated charge transfer. Nanoscale Res. Lett..

[50] Lee W.H., Suk J.W., Lee J., Hao Y., Park J., Yang J.W., Ha H.-W., Murali S., Chou H., Akinwande D. (2012). Simultaneous transfer and doping of CVD-grown graphene by fluoropolymer for transparent conductive films on plastic. ACS Nano.

[51] Pandey S.K., Alsalman H., Azadani J.G., Izquierdo N., Low T., Campbell S.A. (2018). Controlled p-type substitutional doping in large-area monolayer WSe_2_ crystals grown by chemical vapor deposition. Nanoscale.

[52] Zhang K., Bersch B.M., Joshi J., Addou R., Cormier C.R., Zhang C., Xu K., Briggs N.C., Wang K., Subramanian S. (2018). Tuning the electronic and photonic properties of monolayer MoS_2_ via in situ rhenium substitutional doping. Adv. Funct. Mater..

[53] Kim Y., Bark H., Kang B., Lee C. (2019). Wafer-scale substitutional doping of monolayer MoS_2_ films for high-performance optoelectronic devices. ACS Appl. Mater. Interfaces.

[54] Yue Y., Jiang C., Han Y., Wang M., Ren J., Wu Y. (2020). Magnetic anisotropies of Mn-, Fe-, and Co-doped monolayer MoS_2_. J. Magn. Magn. Mater..

[55] Jin Y., Zeng Z., Xu Z., Lin Y.-C., Bi K., Shao G., Hu T.S., Wang S., Li S., Suenaga K. (2019). Synthesis and transport properties of degenerate p-type Nb-doped WS_2_ monolayers. Chem. Mater..

[56] Zhang P., Cheng N., Li M., Zhou B., Bian C., Wei Y., Wang X., Jiang H., Bao L., Lin Y. (2020). Transition-Metal Substitution-Induced Lattice Strain and Electrical Polarity Reversal in Monolayer WS_2_. ACS Appl. Mater. Interfaces.

[57] Chang R.-J., Sheng Y., Ryu G.H., Mkhize N., Chen T., Lu Y., Chen J., Lee J.K., Bhaskaran H., Warner J.H. (2019). Postgrowth Substitutional Tin Doping of 2D WS2 Crystals Using Chemical Vapor Deposition. ACS Appl. Mater. Interfaces.

[58] Gao H., Suh J., Cao M.C., Joe A.Y., Mujid F., Lee K.-H., Xie S., Poddar P., Lee J.-U., Kang K. (2020). Tuning electrical conductance of MoS_2_ monolayers through substitutional doping. Nano Lett..

[59] Zhang T., Fujisawa K., Zhang F., Liu M., Lucking M.C., Gontijo R.N., Lei Y., Liu H., Crust K., Granzier-Nakajima T. (2020). Universal in situ substitutional doping of transition metal dichalcogenides by liquid-phase precursor-assisted synthesis. ACS Nano.

[60] Kanade C., Arbuj S., Kanade K., Kim K.S., Yeom G.Y., Kim T., Kale B. (2018). Hierarchical nanostructures of nitrogen-doped molybdenum sulphide for supercapacitors. RSC Adv..

[61] Zhao B., Liu L., Cheng G., Li T., Qi N., Chen Z., Tang Z. (2017). Interaction of O_2_ with monolayer MoS_2_: Effect of doping and hydrogenation. Mater. Des..

[62] Yang L., Majumdar K., Liu H., Du Y., Wu H., Hatzistergos M., Hung P., Tieckelmann R., Tsai W., Hobbs C. (2014). Chloride molecular doping technique on 2D materials: WS_2_ and MoS_2_. Nano Lett..

[63] Tang B., Yu Z.G., Huang L., Chai J., Wong S.L., Deng J., Yang W., Gong H., Wang S., Ang K.-W. (2018). Direct n-to p-type channel conversion in monolayer/few-layer WS_2_ field-effect transistors by atomic nitrogen treatment. ACS Nano.

[64] Kim K.H., Kim K.S., Ji Y.J., Moon I., Heo K., Kang D.-H., Kim K.N., Yoo W.J., Park J.-H., Yeom G.Y. (2020). Effect of large work function modulation of MoS 2 by controllable chlorine doping using a remote plasma. J. Mater. Chem. C.

[65] Liang Q., Gou J., Zhang Q., Zhang W., Wee A.T.S. (2020). Oxygen-induced controllable p-type doping in 2D semiconductor transition metal dichalcogenides. Nano Res..

[66] Komsa H.-P., Kotakoski J., Kurasch S., Lehtinen O., Kaiser U., Krasheninnikov A.V. (2012). Two-dimensional transition metal dichalcogenides under electron irradiation: Defect production and doping. Phys. Rev. Lett..

[67] Kang D.-H., Dugasani S.R., Park H.-Y., Shim J., Gnapareddy B., Jeon J., Lee S., Roh Y., Park S.H., Park J.-H. (2016). Ultra-low doping on two-dimensional transition metal dichalcogenides using DNA nanostructure doped by a combination of lanthanide and metal ions. Sci. Rep..

[68] Du Y., Liu H., Neal A.T., Si M., Peide D.Y. (2013). Molecular Doping of Multilayer MOS_2_ Field-Effect Transistors: Reduction in Sheet and Contact Resistances. IEEE Electron. Device Lett..

[69] Lockhart de la Rosa C.J., Phillipson R., Teyssandier J., Adisoejoso J., Balaji Y., Huyghebaert C., Radu I., Heyns M., De Feyter S., De Gendt S. (2016). Molecular doping of MoS_2_ transistors by self-assembled oleylamine networks. Appl. Phys. Lett..

[70] Bertolazzi S., Gobbi M., Zhao Y., Backes C., Samorì P. (2018). Molecular chemistry approaches for tuning the properties of two-dimensional transition metal dichalcogenides. Chem. Soc. Rev..

[71] Xu K., Wang Y., Zhao Y., Chai Y. (2017). Modulation doping of transition metal dichalcogenide/oxide heterostructures. J. Mater. Chem. C.

[72] Cai Y., Zhou H., Zhang G., Zhang Y.-W. (2016). Modulating carrier density and transport properties of MoS_2_ by organic molecular doping and defect engineering. Chem. Mater..

[73] Cho Y., Park J.H., Kim M., Jeong Y., Yu S., Lim J.Y., Yi Y., Im S. (2019). Impact of organic molecule-induced charge transfer on operating voltage control of both n-MoS_2_ and p-MoTe_2_ transistors. Nano Lett..

[74] Lim J.Y., Pezeshki A., Oh S., Kim J.S., Lee Y.T., Yu S., Hwang D.K., Lee G.H., Choi H.J., Im S. (2017). Homogeneous 2D MoTe2 p–n Junctions and CMOS Inverters formed by Atomic-Layer-Deposition-Induced Doping. Adv. Mater..

[75] Fang H., Tosun M., Seol G., Chang T.C., Takei K., Guo J., Javey A. (2013). Degenerate n-doping of few-layer transition metal dichalcogenides by potassium. Nano Lett..

[76] Sim D.M., Kim M., Yim S., Choi M.-J., Choi J., Yoo S., Jung Y.S. (2015). Controlled doping of vacancy-containing few-layer MoS_2_ via highly stable thiol-based molecular chemisorption. ACS Nano.

[77] Benjamin C.J., Zhang S., Chen Z. (2018). Controlled doping of transition metal dichalcogenides by metal work function tuning in phthalocyanine compounds. Nanoscale.

[78] Mouri S., Miyauchi Y., Matsuda K. (2013). Tunable photoluminescence of monolayer MoS_2_ via chemical doping. Nano Lett..

[79] Kang D.-H., Shim J., Jang S.K., Jeon J., Jeon M.H., Yeom G.Y., Jung W.-S., Jang Y.H., Lee S., Park J.-H. (2015). Controllable nondegenerate p-type doping of tungsten diselenide by octadecyltrichlorosilane. ACS Nano.

[80] Hong S., Kim K.L., Cho Y., Cho H., Park J.H., Park C., Im S. (2020). Complementary Type Ferroelectric Memory Transistor Circuits with P-and N-Channel MoTe2. Adv. Electron. Mater..

[81] Min S.-W., Yoon M., Yang S.J., Ko K.R., Im S. (2018). Charge-transfer-induced p-type channel in MoS_2_ flake field effect transistors. ACS Appl. Mater. Interfaces.

[82] Kang Y., Han S. (2017). An origin of unintentional doping in transition metal dichalcogenides: The role of hydrogen impurities. Nanoscale.

[83] Fan S., Tang X., Zhang D., Hu X., Liu J., Yang L., Su J. (2019). Ambipolar and n/p-type conduction enhancement of two-dimensional materials by surface charge transfer doping. Nanoscale.

[84] Jo S.H., Kang D.H., Shim J., Jeon J., Jeon M.H., Yoo G., Kim J., Lee J., Yeom G.Y., Lee S. (2016). A High-Performance WSe_2_/h-BN Photodetector using a Triphenylphosphine (PPh3)-Based n-Doping Technique. Adv. Mater..

[85] Hong S., Yoo G., Kim D.H., Song W.G., Le O.K., Hong Y.K., Takahashi K., Omkaram I., Son D.N., Kim S. (2017). The doping mechanism and electrical performance of polyethylenimine-doped MoS_2_ transistor. Phys. Status Solidi C.

[86] Najmaei S., Zou X., Er D., Li J., Jin Z., Gao W., Zhang Q., Park S., Ge L., Lei S. (2014). Tailoring the physical properties of molybdenum disulfide monolayers by control of interfacial chemistry. Nano Lett..

[87] Heo K., Jo S.-H., Shim J., Kang D.-H., Kim J.-H., Park J.-H. (2018). Stable and reversible triphenylphosphine-based n-type doping technique for molybdenum disulfide (MoS_2_). ACS Appl. Mater. Interfaces.

[88] Jing Y., Tang Q., He P., Zhou Z., Shen P. (2015). Small molecules make big differences: Molecular doping effects on electronic and optical properties of phosphorene. Nanotechnology.

[89] Hong S., Choi S.H., Park J., Yoo H., Oh J.Y., Hwang E., Yoon D.H., Kim S. (2020). Sensory Adaptation and Neuromorphic Phototransistors Based on CsPb (Br1–x I x) 3 Perovskite and MoS_2_ Hybrid Structure. ACS Nano.

[90] Ali M.H., Kang D.-H., Park J.-H. (2018). Rhenium diselenide (ReSe2) infrared photodetector enhanced by (3-aminopropyl) trimethoxysilane (APTMS) treatment. Org. Electron..

[91] Hong S., Im H., Hong Y.K., Liu N., Kim S., Park J.H. (2018). n-Type Doping Effect of CVD-Grown Multilayer MoSe2 Thin Film Transistors by Two-Step Functionalization. Adv. Electron. Mater..

[92] Zhang S.N., Benjamin C.J., Chen Z. Molecular Doping of Transition Metal Dichalcogenides Using Metal Phythalocyanines. Proceedings of the 2017 75th Annual Device Research Conference (DRC).

[93] Yoo H., Hong S., Moon H., On S., Ahn H., Lee H.K., Kim S., Hong Y.K., Kim J.J. (2018). Chemical doping effects on CVD-grown multilayer MoSe2 transistor. Adv. Electron. Mater..

[94] Yoo H., Hong S., On S., Ahn H., Lee H.-K., Hong Y.K., Kim S., Kim J.-J. (2018). Chemical doping effects in multilayer MoS_2_ and its application in complementary inverter. ACS Appl. Mater. Interfaces.

[95] Park H.-Y., Dugasani S.R., Kang D.-H., Jeon J., Jang S.K., Lee S., Roh Y., Park S.H., Park J.-H. (2014). n-and p-type doping phenomenon by artificial DNA and M-DNA on two-dimensional transition metal dichalcogenides. ACS Nano.

[96] Jo S.H., Park H.Y., Kang D.H., Shim J., Jeon J., Choi S., Kim M., Park Y., Lee J., Song Y.J. (2016). Broad detection range rhenium diselenide photodetector enhanced by (3-aminopropyl) triethoxysilane and triphenylphosphine treatment. Adv. Mater..

[97] Nakano M., Osaka I., Takimiya K. (2017). Control of Major Carriers in an Ambipolar Polymer Semiconductor by Self-Assembled Monolayers. Adv. Mater..

[98] Weitz R.T., Zschieschang U., Effenberger F., Klauk H., Burghard M., Kern K. (2007). High-performance carbon nanotube field effect transistors with a thin gate dielectric based on a self-assembled monolayer. Nano Lett..

[99] Lv Y., Yao L., Gu C., Xu Y., Liu D., Lu D., Ma Y. (2011). Electroactive Self-Assembled Monolayers for Enhanced Efficiency and Stability of Electropolymerized Luminescent Films and Devices. Adv. Funct. Mater..

[100] Klauk H., Zschieschang U., Pflaum J., Halik M. (2007). Ultralow-power organic complementary circuits. Nature.

[101] Pernstich K., Haas S., Oberhoff D., Goldmann C., Gundlach D., Batlogg B., Rashid A., Schitter G. (2004). Threshold voltage shift in organic field effect transistors by dipole monolayers on the gate insulator. J. Appl. Phys..

[102] Zhu X., Li D., Liang X., Lu W.D. (2019). Ionic modulation and ionic coupling effects in MoS_2_ devices for neuromorphic computing. Nat. Mater..

[103] Feng S., Lin Z., Gan X., Lv R., Terrones M. (2017). Doping two-dimensional materials: Ultra-sensitive sensors, band gap tuning and ferromagnetic monolayers. Nanoscale Horiz..

[104] Choi S.Y., Kim Y., Chung H.-S., Kim A.R., Kwon J.-D., Park J., Kim Y.L., Kwon S.-H., Hahm M.G., Cho B. (2017). Effect of Nb doping on chemical sensing performance of two-dimensional layered MoSe2. ACS Appl. Mater. Interfaces.

[105] Sarkar D., Xie X., Kang J., Zhang H., Liu W., Navarrete J., Moskovits M., Banerjee K. (2015). Functionalization of transition metal dichalcogenides with metallic nanoparticles: Implications for doping and gas-sensing. Nano Lett..

[106] Lv R., Li Q., Botello-Méndez A.R., Hayashi T., Wang B., Berkdemir A., Hao Q., Elías A.L., Cruz-Silva R., Gutiérrez H.R. (2012). Nitrogen-doped graphene: Beyond single substitution and enhanced molecular sensing. Sci. Rep..

[107] Sharma A., Khan M.S., Husain M., Khan M.S., Srivastava A. (2018). Sensing of CO and NO on Cu-doped MoS_2_ monolayer-based single electron transistor: A first principles study. IEEE Sens. J..

[108] Suvansinpan N., Hussain F., Zhang G., Chiu C.H., Cai Y., Zhang Y.-W. (2016). Substitutionally doped phosphorene: Electronic properties and gas sensing. Nanotechnology.

[109] Cho S.-Y., Koh H.-J., Yoo H.-W., Kim J.-S., Jung H.-T. (2017). Tunable volatile-organic-compound sensor by using Au nanoparticle incorporation on MoS_2_. ACS Sens..

[110] Choi G.J., Baek S.H., Lee S.S., Khan F., Kim J.H., Park I.K. (2019). Performance enhancement of triboelectric nanogenerators based on polyvinylidene fluoride/graphene quantum dot composite nanofibers. J. Alloys Compd..

[111] Chen H., Zhang S., Zou Y., Zhang C., Zheng B., Huang C., Zhang B., Xing C., Xu Y., Wang J. (2020). Performance-Enhanced Flexible Triboelectric Nanogenerator Based on Gold Chloride-Doped Graphene. ACS Appl. Electron. Mater..

[112] Zhao L., Chen K., Yang F., Zheng M., Guo J., Gu G., Zhang B., Qin H., Cheng G., Du Z. (2019). The novel transistor and photodetector of monolayer MoS_2_ based on surface-ionic-gate modulation powered by a triboelectric nanogenerator. Nano Energy.

[113] Gupta A., Sakthivel T., Seal S. (2015). Recent development in 2D materials beyond graphene. Prog. Mater. Sci..

